# Response of Blood Perfusion at ST 36 Acupoint after Drinking Cold Glucose or Saline Injection

**DOI:** 10.1155/2017/4212534

**Published:** 2017-03-30

**Authors:** Guangjun Wang, Shuyong Jia, Hongyan Li, Ze Wang, Yuying Tian, Weibo Zhang

**Affiliations:** Institute of Acupuncture and Moxibustion, China Academy of Chinese Medical Sciences, Beijing, China

## Abstract

Skin blood flux (SkBF) changes caused by drinking cold water are generally associated with vagal tone and osmotic factors in digestive system. According to acupuncture theory, change of SkBF at ST 36 might reflect the functional changes of digestive system. The aim of this study is to analyze the changes of SkBF after drinking 3°C 0.9% saline or 5% glucose injection by monitor blood flux at bilateral ST 36. The results indicated that, after drinking different cold water, the change ratio of SkBF at right side ST 36 has been different. Because all solutions have the same temperature (3°C) and both saline and glucose solution have the same osmolality, suggesting that the SkBF changes resulting from drinking cold water are not regulated just by the vagal tone and osmolality, there must have been other factors. These results have not been consistent with the frequency domain results of heart rate variability (HRV) analysis. Coherence analysis of blood flux signals at bilateral ST 36 indicated that there have been different coherence-frequency curves among different groups in special frequency bands, which suggested that coherence analysis might provide a potential tool to evaluate different status.

## 1. Background

Acupuncture has been widely used to treatment diseases in clinical practice at least for 2500 years. During the past 30 years, scientists have demonstrated the neurobiological basis for many acupuncture effects [1], which has made it more acceptable. In traditional acupuncture theory, reasonable choice of acupoints is the basis for treatment [2, 3]. Zusanli acupoint (ST 36) belongs to stomach meridian and is commonly used point to treat digestive system diseases. This view has been confirmed not only from clinical study [4, 5], but also from the basic research [6, 7].

According to Traditional Chinese Medical (TCM) theory, the acupuncture effect was based on the normal flow of Qi-blood along meridians. Compared to the concept of Qi, blood might be easy to understand. Thus, the relationship of acupuncture and circulation has been focused on recently [8–10]. Furthermore, some studies have suggested that the skin microvasculature mirrors the vascular function of other parts of the body [11–13], which is in line with the basic standpoints of acupuncture theory. Based on the above understanding, blood perfusion in ST 36 might be as a potential index to evaluate stomach functional activity after stimulation.

Despite humans preferring to drink water at a temperature below usual room temperatures [14], studies addressing the role of water temperature on blood flux changes are scarce. There are only a few reports where the effects of water temperature were investigated in healthy humans [15, 16], and the results indicated that ingestion of water activates distinct gastrointestinal vagal afferent nerve fibers in a temperature-dependent manner and could influence subsequent cardiac vagal tone [17]. On the other hand, scientists suggested that the water effect is not dependent on gastric distension, but relative hypoosmolality of water, which could elicit an autonomic cardiovascular response in humans through osmoreceptive nerve fibers in the gut or portal circulation [18, 19]. However, previous study indicated that oral glucose intake inhibits hypothalamic neuronal activity more effectively than intravenous (IV) glucose administration does [20], but oral intake of 0.9% saline results in minimal variations in serum albumin, hemoglobin, and hematocrit when compared to IV infusion [21]. These studies show that, in addition to temperature and osmotic pressure mechanism, absorption of glucose or saline in digestive tract might have different autonomic response which resulted in changes of peripheral blood perfusion in systemic regulation. Therefore, we hypothesized that ingestion of different cold injection influences human cutaneous blood perfusion, especially around ST 36, although they have the same osmotic pressure and temperature. To test these hypotheses, we compared, on a randomized control design, the cutaneous blood perfusion changes in response to different cold injection, such as glucose, saline, and control of mineral water, and evaluated additionally their potentially differential impacts on cardiovascular variables.

## 2. Methods

### 2.1. Inclusion and Exclusion Criteria

Eligible subjects had to be heathy and between the ages of 18 and 60 years. All participants were requested to avoid alcohol, tea, or coffee for at least 24 hours prior to the test. None of the subjects had any diseases or were taking any medication affecting cardiovascular or autonomic regulation. If an abnormal electrocardiogram is found in the measurement, the subject is excluded.

### 2.2. Participants and Groups

**Figure 1:**
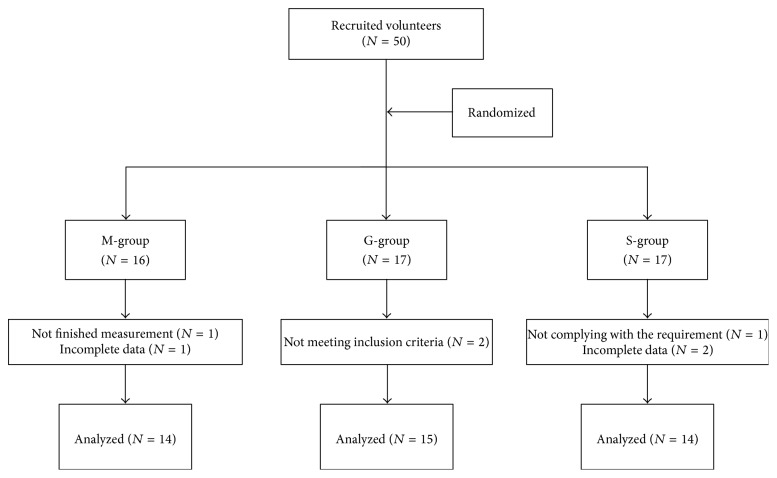
Flow diagram of participants in the study.

A total of 50 healthy subjects were enrolled in the study, and a total of 43 subjects completed all of the measurements and included statistical analysis (Figure 1). The general characters are presented in Table 1. Sample size was determined based on the studies where healthy humans were investigated to analyze water ingestion response [17, 22].

All experiments took place in a quiet, temperature controlled (24–26°C) laboratory and started between 08:00 and 09:00. On arrival at the laboratory, subjects were asked to empty their bladders. Following a period for cardiovascular stability (40 min), a baseline recording was then made for 8 min. Then, the test subjects ingested over 5 min, either 500 mL of cold (3°C) 0.9% saline injection (S-group), 5% glucose injection, or mineral water (M-group); ECG and skin blood flux were monitored.

### 2.3. Protocol for Measurement of Blood Perfusion

Both legs were exposed and bilateral Zusanli acupoints (ST 36) were marked by senior acupuncture doctor. Blood perfusion signals were recorded using PeriFlux System 5000 (Perimed AB, Stockholm, Sweden) with 64 Hz sample rate and 0.2 s time constant. An optical fiber probe connected with the PeriFlux 5000 was used to illuminate and collect the scattered light from the skin tissue. The probe was attached to the surface of interest by means of a two-sided adhesive tape (PF 105-3, Perimed AB, Stockholm, Sweden). In this study, left ST36 was recorded by the first channel and the right ST 36 was recorded by the second channel.

### 2.4. Mean Blood Perfusion Analysis

The recorded file of each subject was opened in the software of PeriSoft for Windows (version 2.5.5, Perimed, Sweden). The detailed data were exported as txt format and then imported to the Matlab software and analyzed. The change ratio of mean blood flux every 8 minutes (totally 30720 points) in either side ST 36 was calculated as

(1) $$\begin{array}{c} \text{change ratio}=\frac{\left(R_{i}-R_{0}\right)}{R_{0}}\times 100. \end{array}$$

### 2.5. Coherence Analysis of Laser Doppler Blood Perfusion Signals

The analysis method can be referenced our previous study [23]. To calculate the coherence between left and the right, the coherence value was estimated by [24, 25]

(2) $$\begin{array}{c} C_{xy}\left(\;f\;\right)=\frac{{\left|P_{xy}\left(f\right)\right|}^{2}}{P_{xx}\left(f\right)P_{yy}\left(f\right)}, \end{array}$$

where *P*_*xy*_(*f*) = ∫_−*∞*_^*∞*^*R*_*xy*_(*t*)*e*^−*jft*^*dt*; thus *P*_*xy*_(*f*) is the Fourier transform of the *R*_*xy*_(*R*_*xy*_ is the cross-correlation of *x* and *y*). Equation (2) obtains the magnitude squared coherence estimate *C*_*xy*_(*f*) of the input signals *x* and *y* using Welch's averaged, modified periodogram method. The value of *C*_*xy*_(*f*) is between 0 and 1 and indicates how well *x* corresponds to *y* at each frequency. Computations were performed by Matlab software (R2011a) through the following script:

(3) Cxy,F=mscoherex,y,window,noverlap,nfft,fs.

In (3), *x* represents the right measurement value of blood perfusion and *y* represents the left side, respectively. Window = hanning(1024), noverlap = 512, nfft = 30000, and fs = 64. *C*_*xy*_ is the coherence value at *F* frequency.

### 2.6. Electrocardiogram Measurement Protocol and HRV Analysis

The analysis method can be referenced in our previous study [26–28]. The ECG recordings were processed with standard II electrocardiographic lead with NeurOne system (NeurOne, MEGA electronics Ltd, Finland). The data were digitized with a sampling rate of 1000 Hz. The raw data was exported with ASC format and then imported into Kubio HRV software and analyzed [29]. The analysis parameter was default. In the frequency domain, the power spectrum density was analyzed with AR spectrum method in normalized units. The very low frequency (VLF) and low frequency (LF) were defined as 0–0.04 Hz and 0.04–0.15 Hz, respectively.

### 2.7. Statistical Analysis

Data are expressed as mean ± SE. The level of significance was defined as *P* < 0.05. Statistical analysis was performed by two-way ANOVA and LSD post hoc test with SPSS software (Version 13.0, SPSS Inc., Chicago, IL). All reported *P* values are two-sided.

## 3. Results

In this study, a total 43 subjects were included in the final statistics (Figure 1). Detailed information of subjects was summarized in Table 1. There were no significant differences in age, height, weight, and body mass index (BMI) among G-group, S-group, and M-group.

### 3.1. Skin Blood Flux

**Figure 2:**
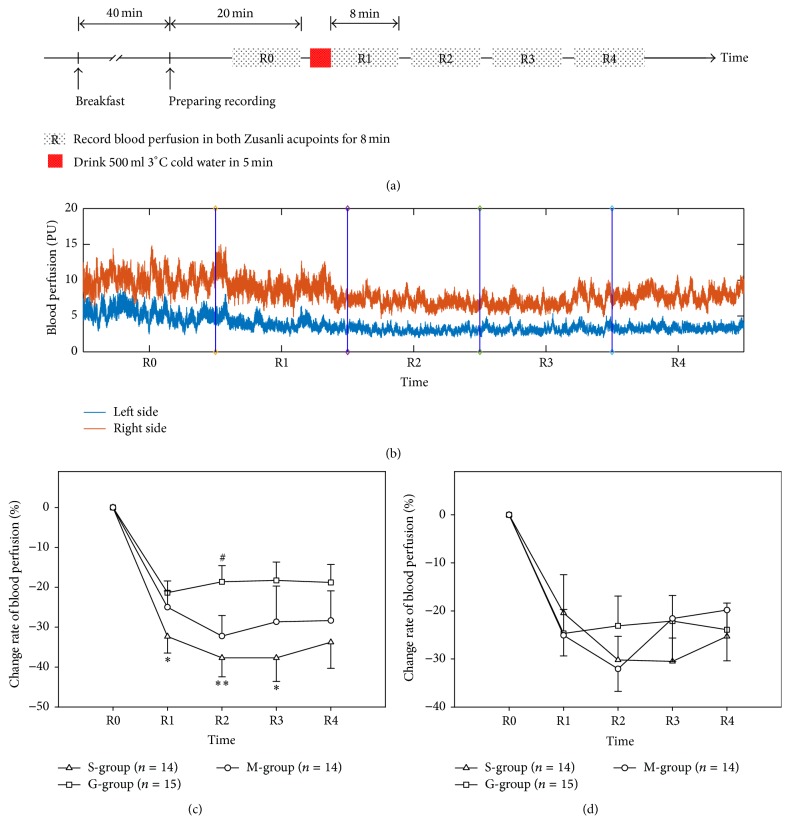
Illustration of the study design (a) and the original blood perfusion signal (b). Time course of the changes in skin blood flux in right ST 36 (c) and left ST 36 (d). ^*∗*^*P* < 0.05; ^*∗∗*^*P* < 0.01; S-group versus G-group; ^#^*P* < 0.05, G-group versus M-group. S-group, saline injection; G-group, glucose injection; M-group, mineral water group. All values are reported as mean ± SE.

The time courses of the responses to the three drinks are shown in Figure 2. Which indicated whether drinking saline, glucose injection, or mineral water and bilateral Zusanli blood flux were decreased obviously after cold stimulation. The results of right ST 36 are shown in Figure 2(c). At the R1 time point, the change ratio of blood perfusion after saline injection drink was −32.32% ± 4.17%, compared with −21.35% ± 2.95% after glucose injection drink and −25.00% ± 4.09% after mineral water drink (ANOVA, *P* = 0.12; LSD post hoc test, S-group versus G-group, *P* = 0.44; G-group versus M-group, *P* = 0.491; S-group versus M-group, *P* = 0.18). At the R2 time point, the change ratio of blood perfusion of right ST 36 after saline injection drink was −37.69% ± 4.74%, compared with −18.61% ± 4.03% after glucose injection drink and −32.23% ± 5.14% after mineral water drink (ANOVA, *P* = 0.016; LSD post hoc test, S-group versus G-group, *P* = 0.006; G-group versus M-group, *P* = 0.043; S-group versus M-group, *P* = 0.415). At the R3 time point, the change ratio of blood perfusion of right ST 36 after saline injection drink was −37.69% ± 5.90%, compared with −18.27% ± 4.59% after glucose injection drink and −28.66% ± 8.98% after mineral water drink (ANOVA, *P* = 0.129; LSD post hoc test, S-group versus G-group, *P* = 0.045; G-group versus M-group, *P* = 0.274; S-group versus M-group, *P* = 0.349). Left side result was shown in Figure 2(d), and there are no significant statistics among three groups.

### 3.2. Coherence Analysis Result

**Figure 3:**
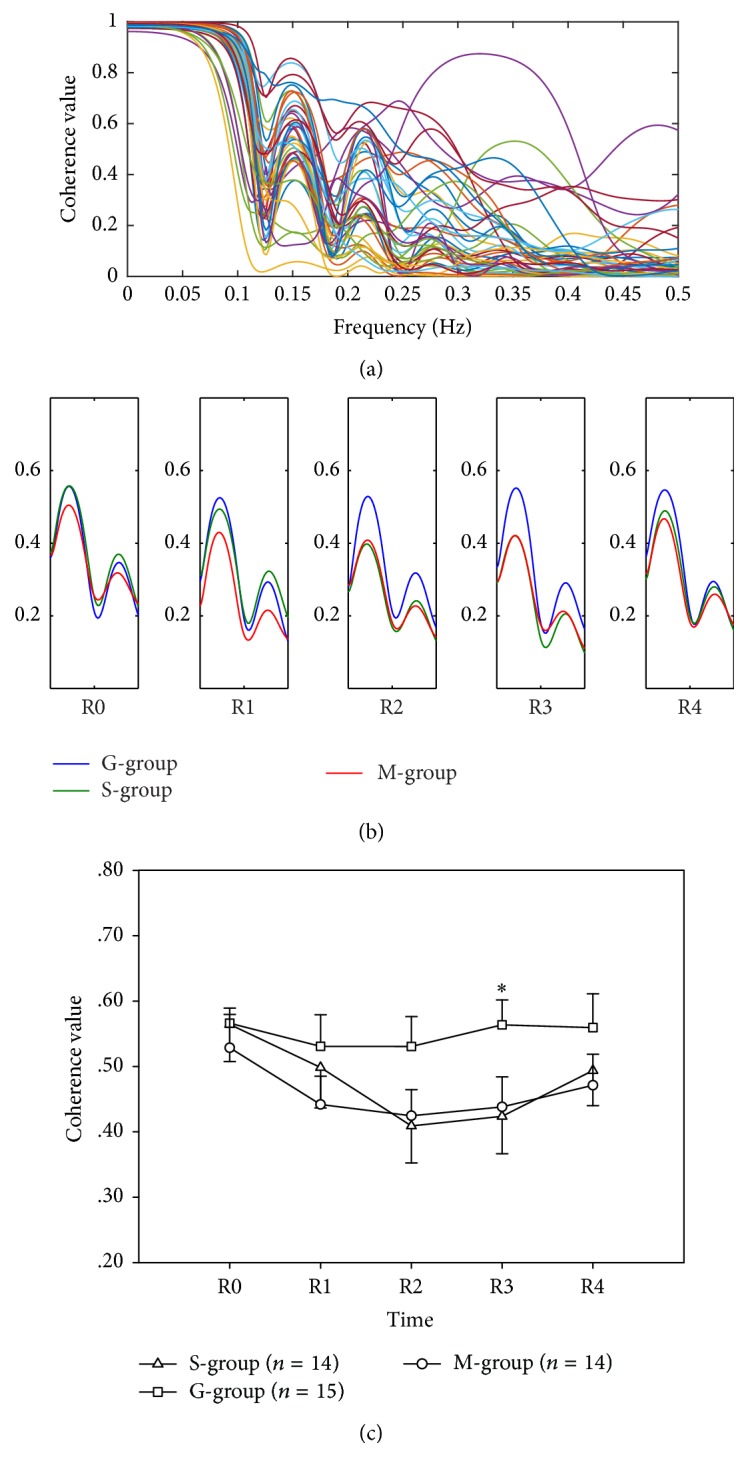
All subjects' (43) coherence value of blood perfusion between left ST 36 and right ST 36 at R0 time point (a). Group average of coherence value from 0.12 Hz to 0.26 Hz (b). Peak value from 0.12 Hz to 0.26 Hz (c). ^*∗*^*P* < 0.05, S-group versus G-group. S-group, saline injection; G-group, glucose injection; M-group, mineral water group. All values are reported as mean ± SE.

The coherence-frequency responses to the three drinks are shown in Figure 3. In the frequency-coherence curve, there is a very clear peak from 0.12 to 0.26 Hz (Figure 2(b)). The group mean of peak value is shown in Figure 2(c). At the R3 time point, the peak value after saline injection drink was 0.42 ± 0.06, compared with 0.56 ± 0.04 after glucose injection drink and 0.44 ± 0.05 after mineral water drink (ANOVA, *P* = 0.079; LSD post hoc test, S-group versus G-group, *P* = 0.042; G-group versus M-group, *P* = 0.067; S-group versus M-group, *P* = 0.832).

### 3.3. Frequency Domain Result of HRV

**Figure 4:**
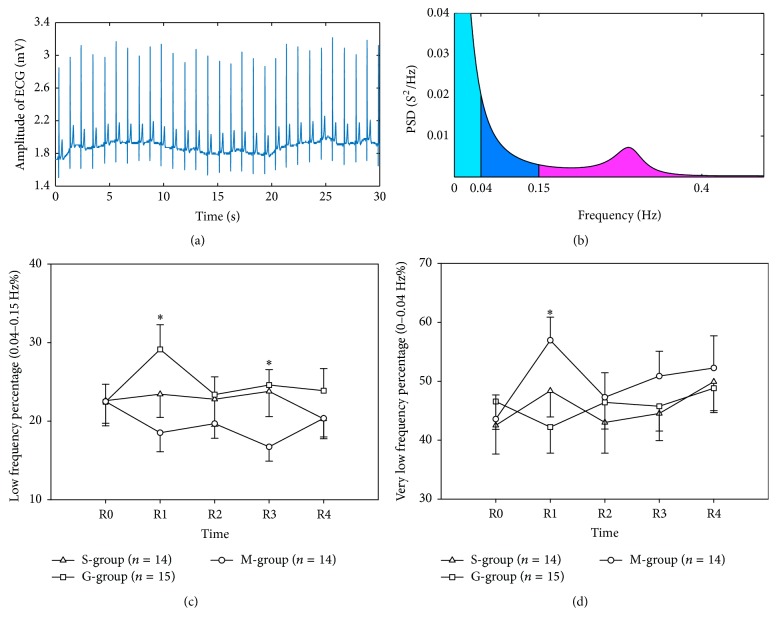
ECG recorded from subject (a). AR spectrum of frequency domain result (b). Difference of low frequency band (0.04–0.15 Hz) and very low frequency band (0–0.04 Hz) was shown in (c) and (d), respectively. ^*∗*^*P* < 0.05, S-group versus M-group. S-group, saline injection; G-group, glucose injection; M-group, mineral water group. All values are reported as mean ± SE.

The frequency domain responses to the three drinks are shown in Figure 4. The low frequency band percentage was shown in Figure 4(c). At the R1 time point, the percentage after saline injection drink was 23.44 ± 2.98, compared with 29.14 ± 3.13 after glucose injection drink and 18.52 ± 2.41 after mineral water drink (ANOVA, *P* = 0.041; LSD post hoc test, S-group versus G-group, *P* = 0.166; G-group versus M-group, *P* = 0.012; S-group versus M-group, *P* = 0.239).

The very low frequency band percentage was shown in Figure 4(d). At the R1 time point, the percentage after saline injection drink was 48.39 ± 4.44, compared with 42.21 ± 4.41 after glucose injection drink and 56.97 ± 3.92 after mineral water drink (ANOVA, *P* = 0.059; LSD post hoc test, S-group versus G-group, *P* = 0.309; G-group versus M-group, *P* = 0.018; S-group versus M-group, *P* = 0.168).

## 4. Discussion

The main finding of this study is there have been different responses of blood perfusion at ST 36 Acupoint after drinking cold glucose or saline. The present study demonstrated that cold injection produced peripheral skin vasoconstriction, suggesting that vasomotor regulation of peripheral tissues might play an important role in cold water digestion. In the peripheral tissues, tiny changes in blood perfusion of cutaneous vasculature can shift blood volumes and cardiovascular control. A transient decrease in the SkBF would produce a relative changes of peripheral sympathetic activation [30]. An explanation can be derived from an activation of thermosensitive afferent vagal nerve fibers which were found in esophagus, stomach, and duodenum [31]. It seems that ingestion of water activates distinct gastrointestinal vagal afferent nerve fibers in a temperature-dependent manner and could influence subsequent cardiac vagal tone [17]. However, our results demonstrated that cold water decreased cutaneous blood flow in the lower extremity, but not in association with a change in the HRV frequency domain result. It has been suggested that cardiac vagal tone is not the only mechanism pathway to explain SkBF response to cold water in young healthy subjects.

Beside cardiac vagal tone, the other factor might be osmolality on autonomic functions [19]. Previous study [18] suggested that water effect is not dependent on gastric distension, but on the water's relative hypoosmolality, which is possibly to elicit an autonomic cardiovascular response in humans [18, 19]. However, in this study, 5% glucose injection and 0.9% saline injection have the same osmolality; it is hard to conclude that the change of sympathetic tone is attributed to water osmolality.

Glucose is commonly used electrolyte and widely used in medical practice. In general, blood glucose levels in the body were regulated by insulin. Previous study indicated that insulin has vasoactive properties [32]. Furthermore, other evidence indicated that insulin causes vasodilatation in the microcirculation in the skin both when given locally and after systemic delivery through an oral glucose load [33]. In this study, the blood perfusion of right ST 36 in G-group is higher than S-group, which might result from insulin action after oral glucose load.

In present study, the change trend of SkBF at bilateral ST 36 is different after drinking different cold water, which is consistent with our previous findings [34, 35]. Previous study have indicated that, in young subjects, sympathetic vasoconstrictor activation after drinking water is not accompanied by an increase in arterial blood pressure [36], which suggested that the change in the vascular tone in the limbs may be partially compensated by opposing changes in other vascular beds [36]. Therefore, it is acceptable that the distribution of the bilateral blood flow and its variation are asymmetric.

Previous studies had indicated that blood flux oscillations at frequencies from 0.0095 to 1.6 Hz might reflect different physiological rhythms [37], which can be separated into five frequency bands in frequency domain [38–41]. Coherence analysis is a method to analysis correlation of two blood flux signals in frequency domain [42]. In previous study, we observed that 30 mmHg inflating occlusion resulted in change of coherence values in middle-aged subjects in special frequency bands; however, the same stimulus did not change the coherence value in young-aged subjects [43] in the same band. Further study indicated that coherence analysis might provide a potential noninvasive method to assess microcirculatory changes in different ages [44]. In present study, the coherence-frequency curves were different after drinking different cold water, especially in the frequency band of 0.12–0.26 Hz, which reflect the activation of intrinsic myogenic and respiration.

## 5. Conclusion

There have been different responses of blood perfusion at ST 36 Acupoint after drinking cold glucose or saline injection, suggesting that the SkBF changes resulting from drinking cold water are not regulated just by the vagal tone and osmolality; there must have been other factors involving these regulations. Coherence analysis result suggested that coherence analysis might provide a potential tool for evaluating cold water load stimulation.

## Figures and Tables

**Table 1:** Subject's gender composition, average age, height, weight, and BMI.

Group	*n*	Gender (female/male)	Age (years, mean ± SD)	Height (cm, mean ± SD)	Weight (kg, mean ± SD)	BMI (mean ± SD)
G-group	15	10/5	26.47 ± 2.39	166.60 ± 5.46	64.33 ± 8.43	23.19 ± 2.93
M-group	14	11/3	25.64 ± 2.47	164.43 ± 8.27	58.71 ± 11.37	21.58 ± 2.70
S-group	14	8/6	25.43 ± 2.53	166.14 ± 6.15	57.50 ± 6.33	20.86 ± 2.30

## Abbreviations

SkBF: Skin blood flux
ST 36: Zusanli acupoint
S-group: Saline injection group
G-group: Glucose injection group
M-group: Mineral water group
ECG: Electrocardiogram
HRV: Heart rate variability
TCM: Traditional Chinese Medicine
BMI: Body mass index
Fig: Figure
SD: Standard deviation
SE: Standard error
ANOVA: Analysis of variance.
